# Monoclinic form of 1,2,4,5-tetra­cyclo­hexyl­benzene

**DOI:** 10.1107/S160053680706758X

**Published:** 2008-01-04

**Authors:** Joel T. Mague, Lisa Linhardt, Iliana Medina, Daniel J. Sattler, Mark J. Fink

**Affiliations:** a Department of Chemistry, Tulane University, New Orleans, LA 70118, USA

## Abstract

The mol­ecule of the title compound, C_30_H_46_, has a crystallographically imposed inversion center and the cyclo­hexyl groups are oriented with their methine H atoms pointing towards one another (H⋯H = 1.99 Å). The cyclohexyl groups adopt chair conformations. A significant C—H⋯π inter­action assembles mol­ecules into layers parallel to (100).

## Related literature

For related structures, see: Mague *et al.* (2008*a* ▶,*b* ▶); Vilardo *et al.* (2000 ▶). For related literature, see: Koudelka *et al.* (1985 ▶); Saito *et al.* (2004 ▶); Schweiger *et al.* (2001 ▶).
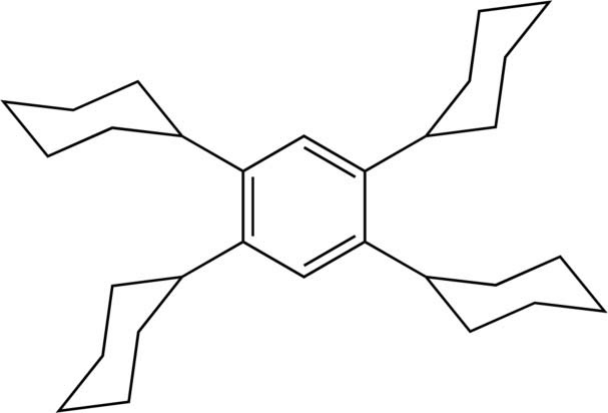


## Experimental

### 

#### Crystal data

C_30_H_46_
*M* *_r_* = 406.67
Monoclinic, 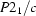

*a* = 10.3868 (7) Å
*b* = 10.1434 (7) Å
*c* = 11.5419 (8) Å
β = 93.314 (1)°
*V* = 1213.99 (14) Å^3^
*Z* = 2
Mo *K*α radiation
μ = 0.06 mm^−1^
*T* = 100 (2) K
0.24 × 0.21 × 0.11 mm

#### Data collection

Bruker SMART APEX CCD area-detector diffractometer
Absorption correction: multi-scan (*SADABS*; Sheldrick, 2002 ▶) *T*_min_ = 0.975, *T*_max_ = 0.993
10421 measured reflections
2805 independent reflections
2460 reflections with *I* > 2σ(*I*)
*R*_int_ = 0.019

#### Refinement

*R*[*F* ^2^ > 2σ(*F* ^2^)] = 0.046
*wR*(*F* ^2^) = 0.126
*S* = 1.04
2805 reflections
136 parameters
H-atom parameters constrained
Δρ_max_ = 0.39 e Å^−3^
Δρ_min_ = −0.21 e Å^−3^

### 

Data collection: *SMART* (Bruker, 2000 ▶); cell refinement: *SAINT-Plus* (Bruker 2004 ▶); data reduction: *SAINT-Plus*; program(s) used to solve structure: *SHELXS97* (Sheldrick, 1997 ▶); program(s) used to refine structure: *SHELXL97* (Sheldrick, 1997 ▶); molecular graphics: *SHELXTL* (Bruker, 2000 ▶); software used to prepare material for publication: *SHELXTL*.

## Supplementary Material

Crystal structure: contains datablocks I, global. DOI: 10.1107/S160053680706758X/gk2127sup1.cif

Structure factors: contains datablocks I. DOI: 10.1107/S160053680706758X/gk2127Isup2.hkl

Additional supplementary materials:  crystallographic information; 3D view; checkCIF report

## Figures and Tables

**Table 1:** Hydrogen-bond geometry (Å, °) *Cg* is the centroid of the aromatic ring.

*D*—H⋯*A*	*D*—H	H⋯*A*	*D*⋯*A*	*D*—H⋯*A*
C6—H6*A*⋯*Cg*^i^	0.99	2.62	3.520 (2)	150

Symmetry code: (i) 
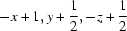
.

**Fig. 1.**
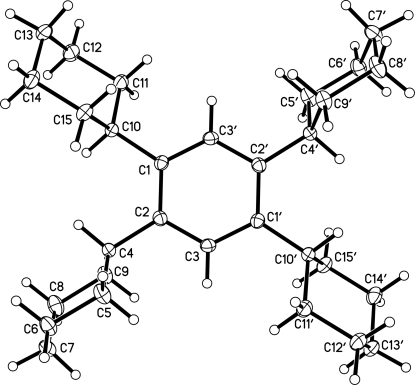
Molecular structure of the title compound. Displacement ellipsoids are drawn at the 50% probability level and H-atoms are represented by spheres of arbitrary radius. Primed atoms are related to unprimed atoms by the symmetry operation 0.5 - x, -y, -z

## supplementary crystallographic information

### Comment

Poly(cyclohexyl)benzenes have been used in the synthesis of a variety of sterically bulky protecting groups (Koudelka *et al.*, 1985); Saito *et al.*, 2004; Vilardo *et al.*, 2000; Schweiger *et al.*, 2001). Crystallization of 1,2,4,5-tetracylohexylbenzene (C_30_H_46_) from hot methylcyclohexane forms colorless needle-shaped crystals together with a smaller quantity having a distinctly different block-shaped morphology. The latter is monoclinic with the molecule having crystallographically imposed centrosymmetry. The cyclohexyl rings adopt the chair conformation and are oriented with their methine hydrogen atoms pointed towards one another (H···H distance 1.99 Å). This contrasts with the structure of 1-bromo-2,4,6-tricyclohexylbenzene (Mague *et al.*, 2008*b*) where the methine hydrogen atoms of the *meta*-disposed cyclohexyl groups point towards the intervening ring substituent. This is presumably to minimize intramolecular contacts between the *ortho*-disposed cyclohexyl rings. Indeed, there are very few close contacts involving these substituents, the shortest being H4···H10 (1.99 Å), H3···H5a (2.18 Å) and H3···H11*a*' (2.07 Å). The plane defined by the atoms C5, C6, C8, C9 ("seat" of the chair) is inclined to the plane of the aromatic ring by 79.0 (2)° while that for the other cyclohexyl ring (C11, C12, C14, C15) is inclined at an angle of only 61.7 (2)°. A significant C—H···π interaction occurs between C6—H6A and the center of gravity (*Cg*) of the aromatic ring in the molecule at 1 - *x*, 1/2 + *y*, 0.5 - *z* where the H—*Cg* distance is 2.62 Å and the C—H···*Cg* angle is 150°. This interaction forms layers of molecules parallel to (100) at approximately *x* = 0.5.

### Experimental

A mixture of chlorocyclohexane (125 ml, 1.05 mol) and benzene (9 ml, 0.1 mol) in a 250 ml 3-necked flask was cooled to -40° C and mechanically stirred while anhydrous AlCl_3_ (6.6 g, 0.05 mol) was added in portions over a 20 min. period. The mixture was allowed to slowly warm to -15° C and the stirring continued for 2 h. The resulting yellow-orange mixture was quenched by pouring it over 800 g of ice and, after thawing, was filtered. The organic layer of the filtrate was separated off, washed several times with water and reduced to a small volume under reduced pressure. After standing for 1 week, a precipitate formed which was filtered off, washed with 10% aqueous HCl and collected by filtration to provide 16.0 g (40%) of white solid (*M*.p. 549–550 K, bulk sample). ^1^H NMR (δ, CDCl_3_): 1.4 (20*H*, br multiplet), 1.8 (20*H*, br multiplet),2.7 (4*H*, multiplet), 7.2 (2*H*, s). ^13^C NMR (δ, CDCl_3_): 26.59, 27.56, 34.97, 123.25, 141.8.

### Refinement

H-atoms were placed in calculated positions (C–H = 0.95 - 0.98 Å) and refined as riding on their carriers with isotropic displacement parameters 1.2 - 1.5 times those of the attached carbon atoms.

### Crystal data

**Table:**

C_30_H_46_	*F*_000_ = 452
*M**_r_* = 406.67	*D*_x_ = 1.113 Mg m^−^^3^
Monoclinic, *P*2_1_/*c*	Mo *K*α radiation λ = 0.71073 Å
Hall symbol: -P 2ybc	Cell parameters from 6595 reflections
*a* = 10.3868 (7) Å	θ = 2.7–28.3º
*b* = 10.1434 (7) Å	µ = 0.06 mm^−^^1^
*c* = 11.5419 (8) Å	*T* = 100 (2) K
β = 93.314 (1)º	Block, colorless
*V* = 1213.99 (14) Å^3^	0.24 × 0.21 × 0.11 mm
*Z* = 2	

### Data collection

**Table:**

Bruker SMART APEX CCD area-detector diffractometer	2805 independent reflections
Radiation source: fine-focus sealed tube	2460 reflections with *I* > 2σ(*I*)
Monochromator: graphite	*R*_int_ = 0.019
Detector resolution: 0 pixels mm^-1^	θ_max_ = 27.6º
*T* = 100(2) K	θ_min_ = 2.0º
ω scans	*h* = −13→13
Absorption correction: multi-scan(SADABS; Sheldrick, 2002)	*k* = −13→13
*T*_min_ = 0.975, *T*_max_ = 0.993	*l* = −14→14
10421 measured reflections	

### Refinement

**Table:**

Refinement on *F*^2^	Secondary atom site location: difference Fourier map
Least-squares matrix: full	Hydrogen site location: inferred from neighbouring sites
*R*[*F*^2^ > 2σ(*F*^2^)] = 0.046	H-atom parameters constrained
*wR*(*F*^2^) = 0.126	*w* = 1/[σ^2^(*F*_o_^2^) + (0.0727*P*)^2^ + 0.3565*P*] where *P* = (*F*_o_^2^ + 2*F*_c_^2^)/3
*S* = 1.04	(Δ/σ)_max_ < 0.001
2805 reflections	Δρ_max_ = 0.39 e Å^−^^3^
136 parameters	Δρ_min_ = −0.21 e Å^−^^3^
Primary atom site location: structure-invariant direct methods	Extinction correction: none

### Special details

**Table:**

Experimental. The diffraction data were collected in three sets of 606 frames (ω scans, 0.3°/scan) at φ settings of 0, 120 and 240°.
Geometry. All e.s.d.'s (except the e.s.d. in the dihedral angle between two l.s. planes) are estimated using the full covariance matrix. The cell e.s.d.'s are taken into account individually in the estimation of e.s.d.'s in distances, angles and torsion angles; correlations between e.s.d.'s in cell parameters are only used when they are defined by crystal symmetry. An approximate (isotropic) treatment of cell e.s.d.'s is used for estimating e.s.d.'s involving l.s. planes.
Refinement. Refinement of *F*^2^ against ALL reflections. The weighted *R*-factor *wR* and goodness of fit *S* are based on *F*^2^, conventional *R*-factors *R* are based on *F*, with *F* set to zero for negative *F*^2^. The threshold expression of *F*^2^ > σ(*F*^2^) is used only for calculating *R*-factors(gt) *etc*. and is not relevant to the choice of reflections for refinement. *R*-factors based on *F*^2^ are statistically about twice as large as those based on *F*, and *R*- factors based on ALL data will be even larger. H-atoms were placed in calculated positions (C—H = 0.95 - 0.98 Å) and included as riding contributions with isotropic displacement parameters 1.2 - 1.5 times those of the attached carbon atoms.

### Fractional atomic coordinates and isotropic or equivalent isotropic displacement parameters (Å^2^)

**Table:**

	*x*	*y*	*z*	*U*_iso_*/*U*_eq_	
C1	0.62897 (10)	−0.01942 (10)	0.04453 (8)	0.0136 (2)	
C2	0.57336 (10)	0.10675 (10)	0.04764 (9)	0.0144 (2)	
C3	0.44681 (10)	0.12214 (10)	0.00191 (9)	0.0152 (2)	
H3	0.4101	0.2079	0.0025	0.018*	
C4	0.64402 (10)	0.22668 (10)	0.09843 (9)	0.0147 (2)	
H4	0.7248	0.1947	0.1408	0.018*	
C5	0.56481 (11)	0.30053 (11)	0.18625 (10)	0.0202 (2)	
H5A	0.4827	0.3308	0.1472	0.024*	
H5B	0.5438	0.2396	0.2494	0.024*	
C6	0.63818 (11)	0.41900 (11)	0.23785 (10)	0.0207 (3)	
H6A	0.5833	0.4663	0.2915	0.025*	
H6B	0.7165	0.3882	0.2829	0.025*	
C7	0.67666 (12)	0.51267 (11)	0.14301 (10)	0.0225 (3)	
H7A	0.7280	0.5861	0.1783	0.027*	
H7B	0.5981	0.5506	0.1034	0.027*	
C8	0.75542 (11)	0.44235 (12)	0.05420 (10)	0.0228 (3)	
H8A	0.8390	0.4141	0.0918	0.027*	
H8B	0.7734	0.5043	−0.0092	0.027*	
C9	0.68369 (11)	0.32171 (11)	0.00324 (9)	0.0185 (2)	
H9A	0.7399	0.2747	−0.0495	0.022*	
H9B	0.6057	0.3512	−0.0429	0.022*	
C10	0.76656 (9)	−0.04649 (10)	0.09169 (9)	0.0142 (2)	
H10	0.8166	0.0370	0.0840	0.017*	
C11	0.83559 (10)	−0.15393 (11)	0.02505 (10)	0.0194 (2)	
H11A	0.7864	−0.2374	0.0283	0.023*	
H11B	0.8383	−0.1277	−0.0574	0.023*	
C12	0.97328 (10)	−0.17627 (12)	0.07605 (10)	0.0225 (3)	
H12A	1.0137	−0.2487	0.0335	0.027*	
H12B	1.0249	−0.0954	0.0661	0.027*	
C13	0.97401 (11)	−0.21099 (11)	0.20452 (11)	0.0223 (3)	
H13A	1.0641	−0.2210	0.2361	0.027*	
H13B	0.9293	−0.2961	0.2140	0.027*	
C14	0.90724 (11)	−0.10416 (12)	0.27195 (10)	0.0211 (3)	
H14A	0.9570	−0.0211	0.2689	0.025*	
H14B	0.9049	−0.1309	0.3543	0.025*	
C15	0.76953 (10)	−0.08069 (11)	0.22168 (9)	0.0176 (2)	
H15A	0.7303	−0.0076	0.2643	0.021*	
H15B	0.7174	−0.1609	0.2327	0.021*	

### Atomic displacement parameters (Å^2^)

**Table:**

	*U*^11^	*U*^22^	*U*^33^	*U*^12^	*U*^13^	*U*^23^
C1	0.0136 (5)	0.0149 (5)	0.0123 (4)	−0.0003 (4)	−0.0001 (4)	0.0019 (4)
C2	0.0159 (5)	0.0138 (5)	0.0134 (5)	−0.0014 (4)	−0.0002 (4)	0.0003 (4)
C3	0.0167 (5)	0.0126 (5)	0.0161 (5)	0.0017 (4)	−0.0004 (4)	0.0011 (4)
C4	0.0148 (5)	0.0121 (5)	0.0169 (5)	−0.0006 (4)	−0.0022 (4)	−0.0004 (4)
C5	0.0215 (5)	0.0203 (5)	0.0192 (5)	−0.0063 (4)	0.0045 (4)	−0.0042 (4)
C6	0.0224 (5)	0.0199 (5)	0.0198 (5)	−0.0033 (4)	0.0025 (4)	−0.0067 (4)
C7	0.0268 (6)	0.0135 (5)	0.0267 (6)	−0.0019 (4)	−0.0032 (5)	−0.0019 (4)
C8	0.0267 (6)	0.0217 (6)	0.0203 (5)	−0.0103 (5)	0.0022 (4)	0.0002 (4)
C9	0.0194 (5)	0.0192 (5)	0.0171 (5)	−0.0044 (4)	0.0017 (4)	−0.0018 (4)
C10	0.0127 (5)	0.0132 (5)	0.0164 (5)	0.0001 (4)	−0.0013 (4)	0.0004 (4)
C11	0.0153 (5)	0.0216 (5)	0.0208 (5)	0.0024 (4)	−0.0012 (4)	−0.0041 (4)
C12	0.0139 (5)	0.0243 (6)	0.0291 (6)	0.0034 (4)	0.0008 (4)	−0.0051 (5)
C13	0.0150 (5)	0.0189 (5)	0.0321 (6)	0.0022 (4)	−0.0052 (4)	0.0022 (5)
C14	0.0178 (5)	0.0249 (6)	0.0198 (5)	0.0013 (4)	−0.0045 (4)	0.0022 (4)
C15	0.0148 (5)	0.0209 (5)	0.0167 (5)	0.0023 (4)	−0.0011 (4)	0.0024 (4)

### Geometric parameters (Å, °)

**Table:**

C1—C3^i^	1.3941 (14)	C8—H8B	0.9900
C1—C2	1.4053 (14)	C9—H9A	0.9900
C1—C10	1.5245 (13)	C9—H9B	0.9900
C2—C3	1.3967 (14)	C10—C11	1.5353 (14)
C2—C4	1.5207 (14)	C10—C15	1.5384 (14)
C3—C1^i^	1.3941 (14)	C10—H10	1.0000
C3—H3	0.9500	C11—C12	1.5316 (14)
C4—C9	1.5357 (14)	C11—H11A	0.9900
C4—C5	1.5368 (14)	C11—H11B	0.9900
C4—H4	1.0000	C12—C13	1.5237 (17)
C5—C6	1.5255 (15)	C12—H12A	0.9900
C5—H5A	0.9900	C12—H12B	0.9900
C5—H5B	0.9900	C13—C14	1.5243 (16)
C6—C7	1.5203 (16)	C13—H13A	0.9900
C6—H6A	0.9900	C13—H13B	0.9900
C6—H6B	0.9900	C14—C15	1.5308 (14)
C7—C8	1.5250 (17)	C14—H14A	0.9900
C7—H7A	0.9900	C14—H14B	0.9900
C7—H7B	0.9900	C15—H15A	0.9900
C8—C9	1.5322 (15)	C15—H15B	0.9900
C8—H8A	0.9900		
			
C3^i^—C1—C2	117.82 (9)	C4—C9—H9A	109.3
C3^i^—C1—C10	119.96 (9)	C8—C9—H9B	109.3
C2—C1—C10	122.21 (9)	C4—C9—H9B	109.3
C3—C2—C1	118.12 (9)	H9A—C9—H9B	107.9
C3—C2—C4	118.62 (9)	C1—C10—C11	113.85 (8)
C1—C2—C4	123.26 (9)	C1—C10—C15	110.75 (8)
C1^i^—C3—C2	124.04 (10)	C11—C10—C15	110.17 (9)
C1^i^—C3—H3	118.0	C1—C10—H10	107.3
C2—C3—H3	118.0	C11—C10—H10	107.3
C2—C4—C9	111.71 (8)	C15—C10—H10	107.3
C2—C4—C5	112.29 (8)	C12—C11—C10	111.42 (9)
C9—C4—C5	110.00 (9)	C12—C11—H11A	109.3
C2—C4—H4	107.5	C10—C11—H11A	109.3
C9—C4—H4	107.5	C12—C11—H11B	109.3
C5—C4—H4	107.5	C10—C11—H11B	109.3
C6—C5—C4	111.54 (9)	H11A—C11—H11B	108.0
C6—C5—H5A	109.3	C13—C12—C11	111.12 (9)
C4—C5—H5A	109.3	C13—C12—H12A	109.4
C6—C5—H5B	109.3	C11—C12—H12A	109.4
C4—C5—H5B	109.3	C13—C12—H12B	109.4
H5A—C5—H5B	108.0	C11—C12—H12B	109.4
C7—C6—C5	110.94 (9)	H12A—C12—H12B	108.0
C7—C6—H6A	109.5	C12—C13—C14	110.84 (9)
C5—C6—H6A	109.5	C12—C13—H13A	109.5
C7—C6—H6B	109.5	C14—C13—H13A	109.5
C5—C6—H6B	109.5	C12—C13—H13B	109.5
H6A—C6—H6B	108.0	C14—C13—H13B	109.5
C6—C7—C8	111.42 (9)	H13A—C13—H13B	108.1
C6—C7—H7A	109.3	C13—C14—C15	111.09 (9)
C8—C7—H7A	109.3	C13—C14—H14A	109.4
C6—C7—H7B	109.3	C15—C14—H14A	109.4
C8—C7—H7B	109.3	C13—C14—H14B	109.4
H7A—C7—H7B	108.0	C15—C14—H14B	109.4
C7—C8—C9	111.33 (9)	H14A—C14—H14B	108.0
C7—C8—H8A	109.4	C14—C15—C10	111.74 (9)
C9—C8—H8A	109.4	C14—C15—H15A	109.3
C7—C8—H8B	109.4	C10—C15—H15A	109.3
C9—C8—H8B	109.4	C14—C15—H15B	109.3
H8A—C8—H8B	108.0	C10—C15—H15B	109.3
C8—C9—C4	111.80 (9)	H15A—C15—H15B	107.9
C8—C9—H9A	109.3		
			
C3^i^—C1—C2—C3	−1.33 (16)	C7—C8—C9—C4	−54.77 (12)
C10—C1—C2—C3	179.74 (9)	C2—C4—C9—C8	−179.76 (9)
C3^i^—C1—C2—C4	178.54 (9)	C5—C4—C9—C8	54.84 (12)
C10—C1—C2—C4	−0.39 (15)	C3^i^—C1—C10—C11	34.20 (13)
C1—C2—C3—C1^i^	1.42 (17)	C2—C1—C10—C11	−146.89 (10)
C4—C2—C3—C1^i^	−178.46 (9)	C3^i^—C1—C10—C15	−90.60 (11)
C3—C2—C4—C9	−73.47 (12)	C2—C1—C10—C15	88.31 (12)
C1—C2—C4—C9	106.67 (11)	C1—C10—C11—C12	179.78 (9)
C3—C2—C4—C5	50.66 (12)	C15—C10—C11—C12	−55.11 (12)
C1—C2—C4—C5	−129.21 (10)	C10—C11—C12—C13	56.53 (13)
C2—C4—C5—C6	178.97 (9)	C11—C12—C13—C14	−56.64 (13)
C9—C4—C5—C6	−55.96 (12)	C12—C13—C14—C15	56.22 (12)
C4—C5—C6—C7	56.89 (12)	C13—C14—C15—C10	−55.83 (12)
C5—C6—C7—C8	−56.00 (12)	C1—C10—C15—C14	−178.25 (9)
C6—C7—C8—C9	54.96 (12)	C11—C10—C15—C14	54.90 (12)

Symmetry codes: (i) −*x*+1, −*y*, −*z*.

### Hydrogen-bond geometry (Å, °)

**Table:**

*D*—H···*A*	*D*—H	H···*A*	*D*···*A*	*D*—H···*A*
C6—H6A···Cg^ii^	0.99	2.62	3.520 (2)	150

Symmetry codes: (ii) −*x*+1, *y*+1/2, −*z*+1/2.
